# Notch3 functions as a regulator of cell self-renewal by interacting with the β-catenin pathway in hepatocellular carcinoma

**DOI:** 10.18632/oncotarget.2898

**Published:** 2015-02-11

**Authors:** Qingyu Zhang, Caijie Lu, Tao Fang, Yongcun Wang, Wenhua Hu, Jie Qiao, Bin Liu, Jie Liu, Nianping Chen, Mingyi Li, Runzhi Zhu

**Affiliations:** ^1^ Laboratory of Hepatobiliary Surgery of Affiliated Hospital of Guangdong Medical College, Zhanjiang Key Laboratory of Hepatobiliary Diseases, Zhanjiang 524001, China; ^2^ Oncology Center, Affiliated Hospital of Guangdong Medical College, Zhanjiang 524001, China; ^3^ Department of Pathology, Affiliated Hospital of Guangdong Medical College, Zhanjiang 524001, China

**Keywords:** Notch3, Beta-catenin, cancer stem cells, hepatocellular carcinoma

## Abstract

The Notch signaling pathway plays a role in cell proliferation, differentiation. Emerging data have revealed aberrant Notch3 expression in hepatocellular carcinoma (HCC). However, whether Notch3 plays a role in tumorigenesis or tumor progression is unclear. In this study, we found that over 71.8% of the cases studied had high Notch3 expression levels (*n* = 32); Notch3 expression positively correlated with alpha-fetoprotein (AFP) levels (*p* = 0.0311) and negatively correlated with the differentiation grade (*p* = 0.042). We demonstrated that the patients with higher levels of Notch3 expression commonly had a poor prognosis. We discovered that Notch3 expression is inversely correlated with β-catenin content but positively associated with the protein level of Nanog. In parallel, we found that Notch3 attenuation resulted in the upregulation of β-catenin and the downregulation of Nanog in the hepatoma cell lines QGY7701 and HepG2. The downregulation of Notch3 enhanced the sensitivity to cisplatin in the QGY7701 and HepG2 cells and inhibited the ability of QGY7701 cells to form tumors. The Notch3-positive cells had higher levels of aldehyde dehydrogenase (ALDH) activity, and a tendency to differentiate into Notch3-negative cells. In conclusion, our study demonstrated that Notch3 plays a role in modulating the stemness of tumor cells via the inactivation of the Wnt/β-catenin pathway.

## INTRODUCTION

Hepatocellular carcinoma (HCC) is the third most common cancer worldwide, and over fifty percent of the cases are found in Asia [1]. More than 80% of HCC patients are diagnosed at advanced stages, by which time, the tumors cannot be surgically removed. However, even when surgical resection is combined with the chemotherapeutic management of HCC, this treatment can still be unsatisfactory due to the recurrence and metastasis of malignant cancer cells. Cancer stem cells (CSCs) or tumor-initiating cells (T-ICs) are the initial cancer cells that possess the properties of tumor progenitor cells, including chemotherapeutic resistance, metastasis and recurrence [2, 3]. Recent evidence suggests that the presence and metastasis of CSCs results in unsatisfactory chemotherapeutic outcomes and poor prognoses [4, 5].

The Notch pathway is a critical signaling pathway that is associated with several physiological processes [6–8]. However, emerging evidence has demonstrated that abnormal Notch signal transduction is implicated in tumorigenesis and progression. The discovery of aberrant Notch gene translocation in human pre-T-cell acute lymphoblastic leukemia (T-ALL) was one of the first indications that the Notch pathway is linked to human cancer pathogenesis [9, 10]. Since then, many studies have demonstrated dysfunctional Notch activity in numerous cancers [11, 12]. The Notch pathway plays a key physiological role in tissue and cell development by regulating the expression of target genes involved in cell proliferation and differentiation [6, 13]. Many studies have proposed that Notch signaling is involved in maintaining the stemness of CSCs. However, other previous studies indicated that the Notch pathway functions as a tumor suppressor in carcinogenesis [11, 14, 15].

Given the uncertainty of whether Notch activity has a positive or negative effect on tumorigenesis and progression, it is important to analyze the function of Notch in specific cancer types and in both clinical and experimental settings. Recently, several studies have proven that the Notch pathway is involved in the progression of HCC, but the mechanism of carcinogenesis underlying HCC initiation is still obscure. Therefore, in this study, we assessed whether the Notch pathway plays a role in the pathological progression of HCC and formulated a hypothesis that Notch participates in the regulation of the CSC population.

## RESULTS

### Notch3 expression is activated in HCC tumor tissues

**Figure 1:**
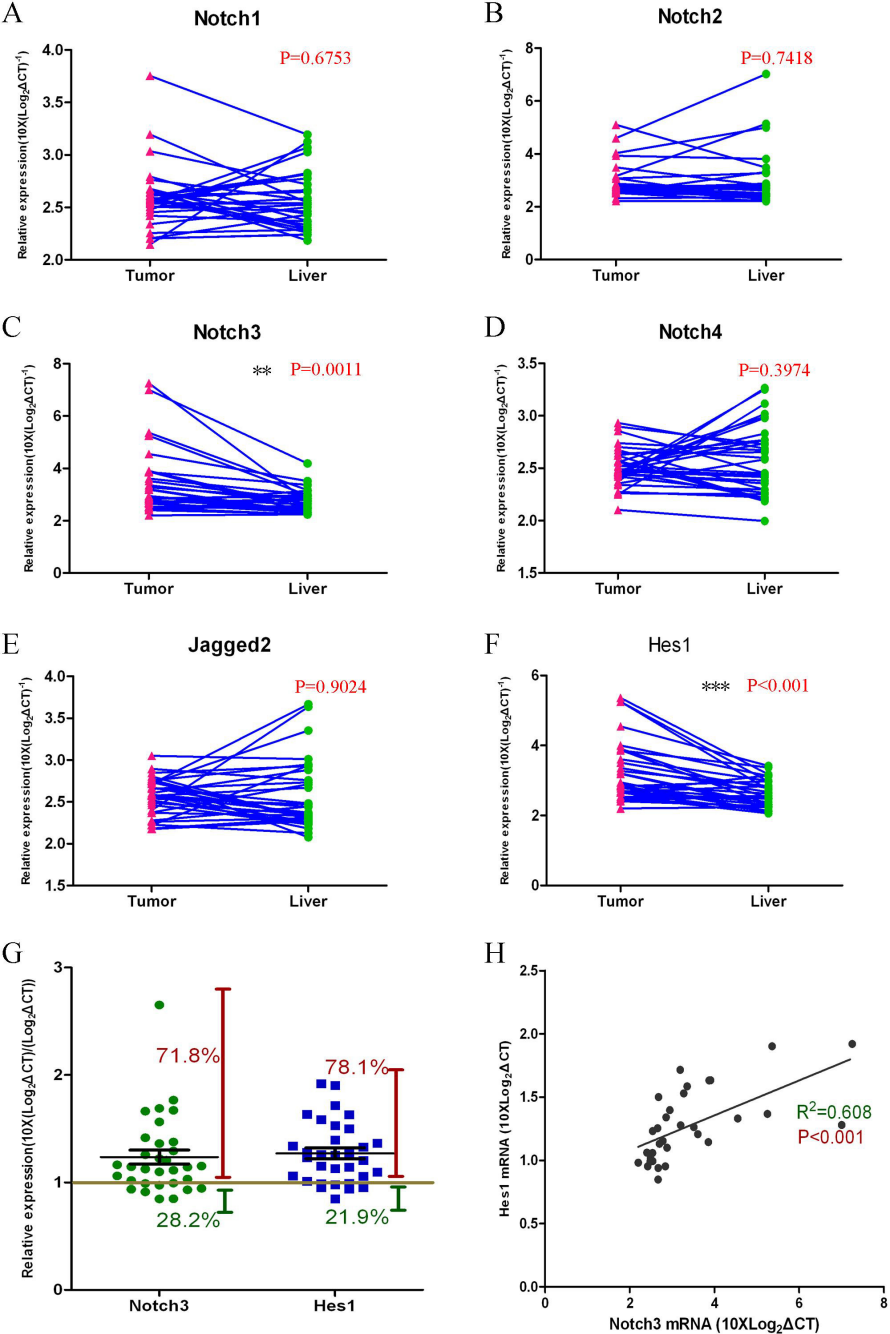
The Notch3 pathway is activated in HCC tumor tissues Gene expression levels were measured with Q-RT-PCR assays **(A–F)**; *Notch3* and *Hes1* genes were expressed at higher levels in tumor tissues compared with normal liver tissues **(C**, *p* = 0.0011, and F, *p* < 0.001; *n* = 32); The expression of other Notch pathway genes (*Notch1*, *Notch2*, *Notch4*, *and Jagged2*) in tumor tissues showed no significant changes compared with normal liver tissues (A, B, D, E; *n* = 32); *Notch3* and *Hes1* gene expression levels were higher in 71.8% and 78.1% of the HCC patients, respectively **(G)**; A Pearson correlation analysis revealed the correlation between *Notch3* and *Hes1* expression **(H**, R^2^ = 0.608, *p* < 0.001).

We assayed the expression profiles of the Notch-related genes (*Notch1–4, Jagged2, Hes1*) in HCC and normal liver tissues with Q-RT-PCR assays (Figure 1A–1F). The *Notch3* and *Hes1* gene expression levels were significantly increased in the tumor tissue compared with the normal liver tissue from the same patient (Figure 1C, 1F). For 71.8% of all the cases tested (*n* = 32), *Notch3* mRNA levels in the tumor were higher than those in the normal liver tissue. In addition, the level of *Hes1* gene expression was increased in tumor tissues over that in normal liver tissues in more than 78.1% of the cases (*n* = 32) (Figure 1G). This positive correlation of *Notch3* and *Hes1* expression (R^2^ = 0.78, *p* < 0.001, *n* = 32) suggests that higher expression levels of *Notch3* in the tumor may result in an upregulation of *Hes1* gene expression (Figure 1H).

### Notch3 expression reflects differentiation properties and correlates with a poor prognosis in HCC

**Figure 2:**
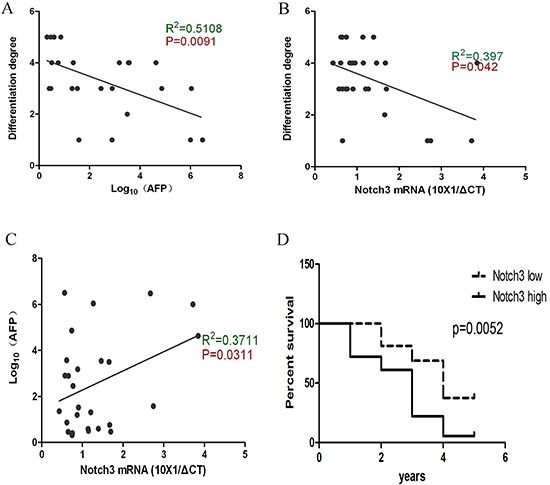
Notch3 reflects the undifferentiated property of HCC cells and is associated with a poor prognosis According to the Pearson correlation analysis, the data demonstrate that the AFP levels are negatively correlated with the degree of differentiation **(A**, *p* = 0.0091); Notch3 is negatively correlated with the degree of differentiation **(B**, *p* = 0.042); Notch3 is positively correlated with the AFP level **(C**, *p* = 0.0311); A high expression of Notch3 indicates a poor outcome after surgical resection **(D**, *p* = 0.0052).

To determine how *Notch3* expression affected the tumor properties, we analyzed the AFP concentration and differentiation grade as a function of *notch*3 levels. The AFP levels negatively correlated with the degree of differentiation, which suggests that AFP is more highly expressed in undifferentiated cells (Figure 2A). We also found that Notch3 expression negatively correlated with the degree of differentiation but positively correlated with AFP concentration (Figure 2B, 2C). The similar correlation of AFP and *Notch*3 with the differentiation state implies that both AFP and Notch3 play roles in maintaining HCC progenitor cells. Furthermore, the survival analysis indicated that Notch3 is a poor prognostic marker in HCC (Figure 2D), which suggests that aberrant Notch3 expression is involved in the progression of the tumor. Based on the results above, it is likely that Notch3 participates in the regulation of the population of CSCs.

### Notch3 activation negatively correlates with the β-catenin signaling pathway

**Figure 3:**
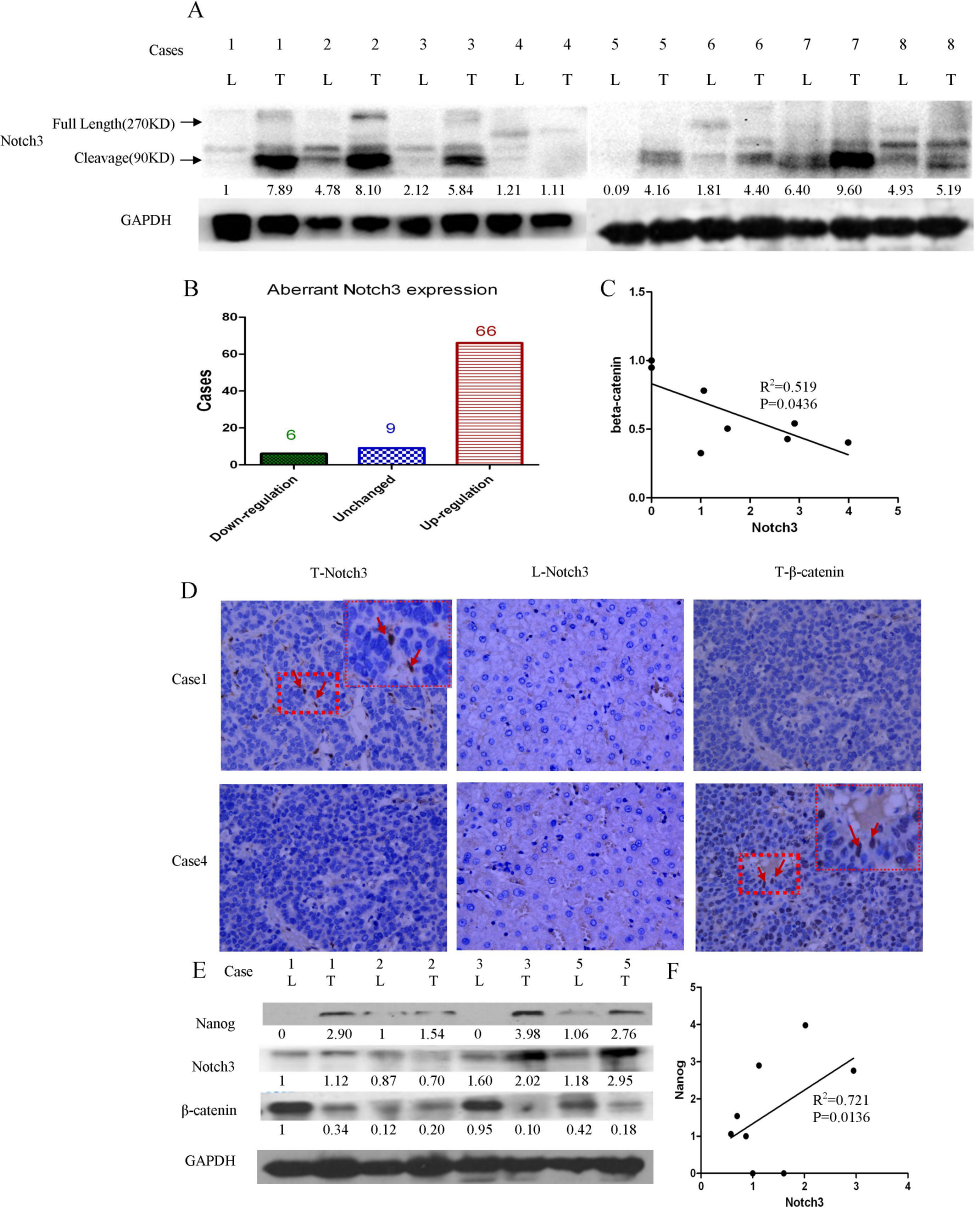
Notch3 signaling activation is negatively associated with β-catenin in HCC tissues Protein levels were detected by western blot analysis. Notch3 proteins were obviously abundant in most of the tumor tissues **(A and B)**; Notch3 expression is negatively correlated with *β-catenin* **(C**, R^2^ = 0.519, *p* < 0.05) and positively correlated with Nanog expression (E, R^2^ = 0.721, *p* < 0.05); The Notch3 and β-catenin proteins were detected by immunohistochemistry **(D)** and western blotting **(E**, L = liver, T = tumor); The correlation between the Notch3 protein level and the Nanog protein level were performed by Pearson correlation analysis **(F**, R^2^ = 0.721, *p* < 0.0136)

β-catenin is implicated in cell-cell adhesion as well as gene transcription. Deregulated β-catenin has been discovered in many types of tumors. The activation of β-catenin promotes tumor proliferation. We questioned whether Notch3 regulates the β-catenin signaling pathway. We determined the Notch3 and β-catenin protein content in the tumor specimens and found Notch3 protein accumulation in the tumor tissues (Figure 3A, 3B); this accumulation was inversely associated with the level of β-catenin (Figure 3C, 3D, 3E). These results suggest that Notch3 might inhibit β-catenin accumulation to modulate tumor cell proliferation. However, previous results have shown that Notch3 expression indicates a poor outcome according to survival analyses. Therefore, we supposed that Notch3 might inhibit β-catenin and result in maintaining the stemness of the tumor cell. We thus detected several stemness-related proteins, including Myc, Oct4 and Nanog. We found that Myc and Oct4 were rarely expressed in the tumor samples. However, a significant amount of Nanog expression was detected in these specimens, which was consistent with the Notch3 expression profile (Figure 3E, 3F). Nanog determines the capacity for cellular self-renewal and proliferation. These data indicated that Notch3 might inhibit β-catenin and increase Nanog to modulate tumor cell differentiation and thereby regulate the CSC population in the pathogenesis of HCC.

### Notch3 plays a crucial role in HCC progression by interacting with the β-catenin pathway and regulating Nanog expression

**Figure 4:**
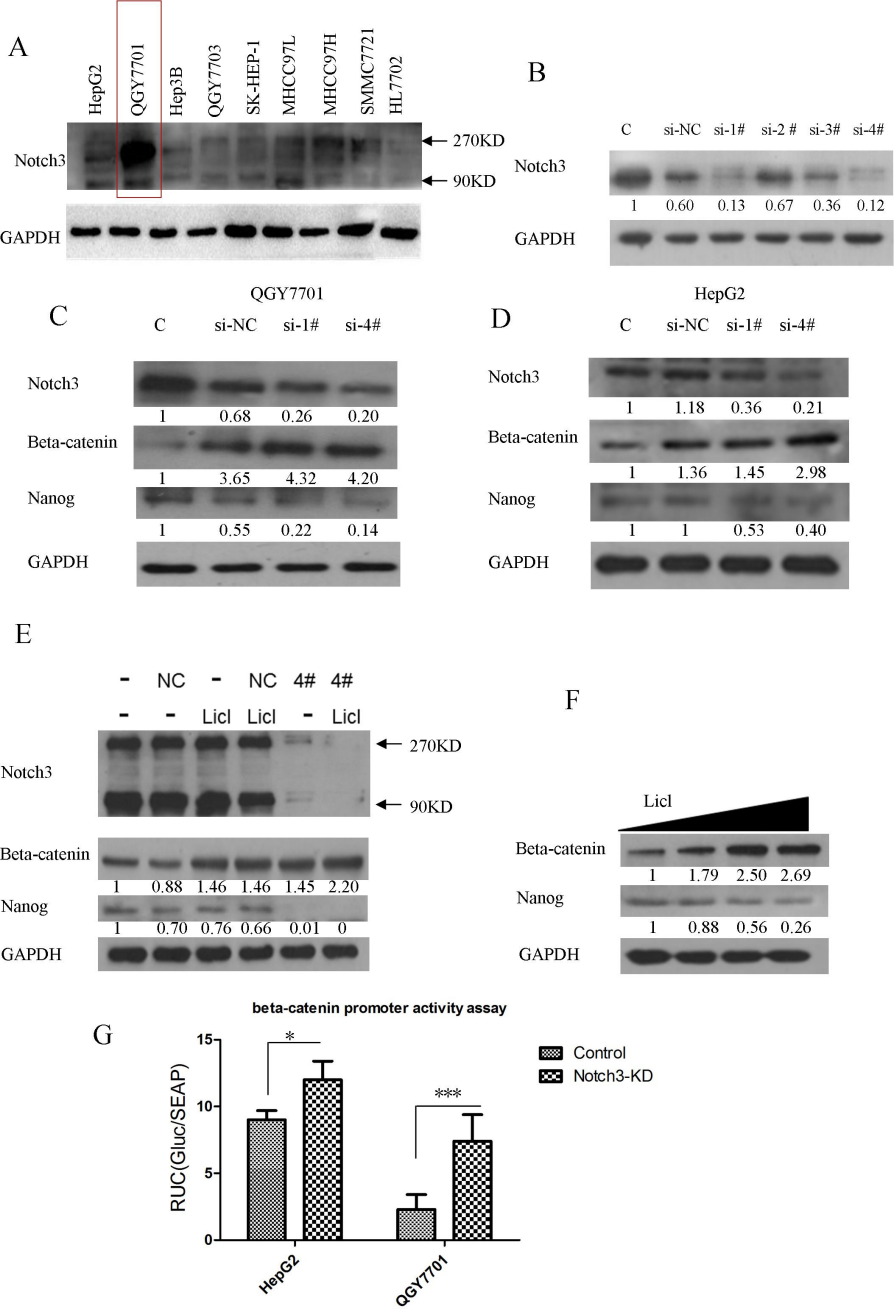
Notch3 plays a crucial role in HCC progression by interacting with β-catenin The Notch3 expression profiles of several HCC cell lines were screened by western blotting **(A)**; siRNA oligos were applied to knockdown Notch3 **(B)**; β-catenin and Nanog were detected via western blotting after Notch3 downregulation by transfection with a siRNA in HepG2 and QGY7701 cells **(C, D)**; The knockdown of Notch3 enhanced the LiCl-mediated activation of the β-catenin pathway and reduced Nanog expression in the QGY7701 cells **(E)**; LiCl-mediated β-catenin activation directly negatively regulated the Nanog protein level in QGY7701 cells **(F)**; A promoter-activity reporter assay was performed to measure the β-catenin promoter activity regulated by Notch3 **(G**, si-NC = si-negative control, si-1# = si-Notch3–1, si-4# = si-Notch3–4).

We also screened the Notch3 protein expression profiles in several hepatoma cell lines. Notch3 exhibited higher expression levels in hepatoma cells compared with the immortalized normal liver cell line HL7702 (Figure 4A). Among the hepatoma cell lines, we found that QGY7701 cells highly express Notch3. Therefore, we silenced Notch3 mRNA expression by siRNA transfection in the QGY7701 cells. We designed 4 pairs of siRNAs for the depletion of Notch3 mRNA, and pairs 1 and 4 were chosen because of their excellent performance regarding Notch3 knockdown (Figure 4B). β-catenin was upregulated and Nanog was downregulated after the depletion of Notch3 via siRNA interference in the QGY7701 and HepG2 cells (Figure 4C, 4D). We further established that Notch3 downregulation negatively regulated β-catenin expression (Figure 4G) and enhanced the activation of β-catenin mediated by LiCl treatment in the QGY7701 cells (Figure 4E). LiCl treatment reduces the expression of Nanog while promoting β-catenin accumulation in the QGY7701 cells (Figure 4F). Notch3 knockdown (lenti-sh-4#) increased the promoter-activity of β-catenin by promoter activity assay (Figure 4F). These data demonstrated that Notch3 negatively regulated β-catenin signaling pathway.

### Notch3 regulates the stemness of cancer cells and affects the differentiation, chemosensitivity and survival of hepatoma cells

**Figure 5:**
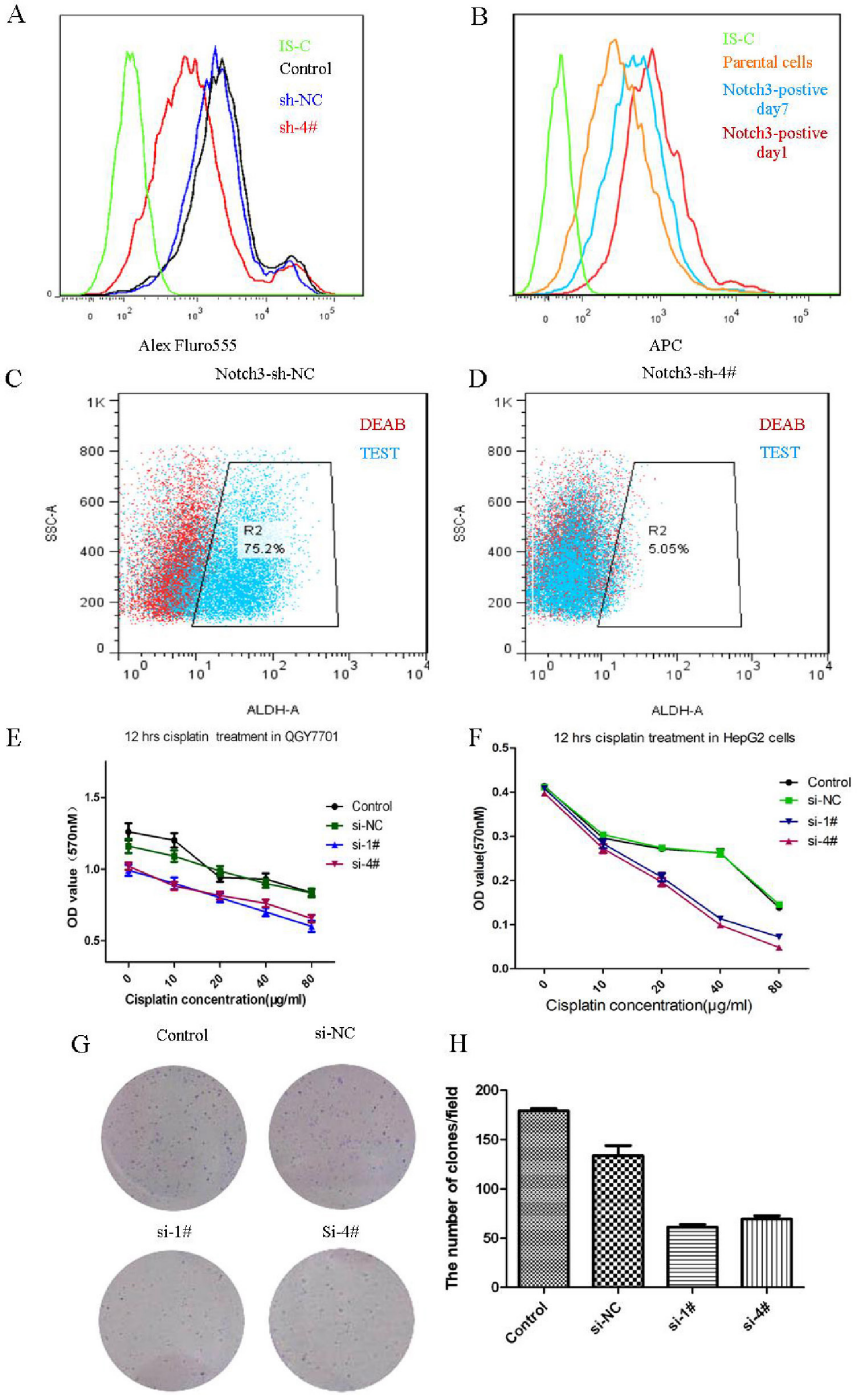
Notch3 regulates the stemness of cancer cells Nanog-positive cells were measured via flow cytometry after the depletion of Notch3 in the QGY7701 cells **(A)**; Notch3-positive cell differentiated into Notch3-negative cells *in vitro* **(B)**; ALDH-positive cells were measured via flow cytometry **(C, D)**; An MTT assay was performed to evaluate the chemosensitivity to cisplatin treatment of the QGY7701 and HepG2 cells **(E, F)**; A colony formation assay was performed to determine the survival of the tumor cells **(G)**; The statistical chart for the colony formation test **(H)**.

We wondered whether Notch3 participated in the regulation of the stemness of CSCs. Nanog is an apparently expressed protein in the Notch3-positive cases. We knocked down Notch3 via siRNA transfection in the QGY7701 cells, and we found that the Nanog-positive cell population was reduced after the Notch3 depletion (Figure 5A). Notch3-positive cells were sorted via FACS, and we found that the Notch3-positive cells gradually lose Notch3 expression after 7 days in culture (Figure 5B). We asked whether Notch3-positive cells definitely have stemness by measuring the level of ALDH activity, which is a property of stem cells. We demonstrated that the QGY7701 cells have a high level of ALDH activity, but this activity was decreased with the knockdown of Notch3 via the interference of a lenti-shRNA (Figure 5C, 5D). The chemosensitivity to cisplatin treatment was assayed with an MTT assay when Notch3 was inhibited by a siRNA in the HepG2 and QGY7701 cells (Figure 5E, 5F). Furthermore, we assayed the ability of the QGY7701 cells to form colonies after Notch3 was downregulated by a siRNA, and we found that Notch3 inhibition reduced the colony-forming capability (Figure 5G, 5H).

## DISCUSSION

CSCs, also called T-ICs, are a highly heterogeneous tumor progenitor cell population that in several carcinomas is involved in the processes of disease progression, including tumor initiation, invasion, metastasis, chemotherapeutic resistance and tumor recurrence [16]. Recently, emerging studies have focused on identifying and characterizing the CSC properties that could be useful targets for tumor prevention [17, 18]. The Notch signaling pathway is a conserved signaling pathway that plays a role in the development of the mammalian body by managing cell fate decisions and proliferation [18]. Previous studies have demonstrated that Notch signaling participates in CSC self-renewal and the maintenance of cancer stemness, as well as tumorigenesis [11, 12]. Our study attempted to explain whether Notch plays a key role in the regulation of hepatocellular CSCs.

Tumor specimens from HCC patients were collected to measure Notch pathway-related genes with Q-RT-PCR assays, which indicated that the levels of Notch3, a Notch receptor family subtype, were increased in tumor tissues. *Hes1*, a Notch pathway target gene [19, 20], was also found to be upregulated in the HCC tumor tissues. We confirmed that *Notch3* gene expression was higher in tumor tissues compared with normal liver tissues in over 70% of all the HCC patients tested. Nearly 80% of the HCC patients also exhibited higher expression levels of *Hes1* in the tumor tissues. These results indicate that Notch3-induced Notch pathway activation is a significant feature of HCC. Our results further confirmed that AFP, a clinical diagnostic marker of HCC, negatively correlates with the degree of differentiation, which was also the case for *Notch3* expression. In addition, *Notch3* expression positively correlated with the AFP levels. However, *Notch3* and AFP co-existed in only a subset of the tumor cell population. Previous studies documented that AFP-positive tumor cells have some properties of HCC CSCs [21, 22]. Therefore, we speculated that the Notch3-positive cells are a subpopulation of CSCs. These results indicate that *Notch3* is expressed in cells that are maintaining in differentiation state. Experienced clinicians frequently find that tumors with higher growth activity seem to be more susceptible to chemotherapeutic treatment. However, HCC cells arrested in the Go phase maintain some CSC properties, such as self-renewal, differentiation capability, tumor recurrence and chemotherapeutic resistance. Thus, Notch3 is likely a distinctive biomarker of CSCs that is relevant to HCC progression.

In this study, we discovered that Notch3 expression is aberrant and confirmed that Notch3 plays an essential role in HCC progression. Hyperactive Notch3 could inhibit the expression of β-catenin and the upregulation of Nanog. β-catenin is a well-known oncogene present in many cancers. The mutation of adenomatous polyposis coli(*APC*) gene, a negative regulator of β-catenin, induces β-catenin activation and the transcription of important target proteins and usually results in cell proliferation and differentiation [23, 24]. According to the expression profiles of Notch3 and β-catenin, it appears that Notch3 plays a paradoxical role in the progression of tumors. However, because Nanog expression is positively correlated with Notch3, it is likely Notch3 functions as a governor to balance tumor cell proliferation and the maintenance of the CSC population by regulating the β-catenin pathway.

We silenced the expression of Notch3 via a siRNA and demonstrated that the downregulation of Notch3 acutely promoted β-catenin expression and enhanced the sensitivity to cisplatin. The downregulation of Notch3 in the QGY7701 cells resulted in the poor survival of tumor cells. The Notch3-knockdown cells had reduced ALDH activity, while the Notch3-positive cells, which were sorted by FACS, were likely to lose Notch3 expression and tended to become similar to the parental cells from which they were selected. We confirmed in this study that Notch3 is potentially a specific marker of HCC and plays a role in the regulation of the CSCs in a hepatoma by interacting with the Wnt/β-catenin pathway. The development of new drugs targeting Notch3 would induce the specific inhibition of the CSC population and promote the regression of HCC.

## MATERIALS AND METHODS

### Clinical specimens

HCC patients participating in this study were diagnosed by an oncologist, and HCC was confirmed by a pathologist. This study was approved by the Medical Ethics committee at the Affiliated Hospital of Guangdong Medical College (Ethics Committee protocol number: PJ2013105). All the participants were informed of how their tissue samples would be used and how the experimental results might be presented. We ensured that all the patients understood this information and signed the written informed consent form. After surgical resection, tumor and normal liver tissues (adjacent non-tumor tissue) were collected immediately and frozen in liquid nitrogen before use.

### Q-RT-PCR

Total RNA was extracted from the tissues with TRIzol reagent. Total RNA (1 μg) was prepared for cDNA production using the PrimeScript^TM^ RT reagent with gDNA eraser (Takara, Dalian, China). Real-time PCR was performed using the SYBR Premix Ex Taq II kit (Takara, Dalian, China). To determine the relative mRNA expression levels, 2X (Log2ΔCT)^−1^ or 10X (ΔCT)^−1^ calculations were used. The 18S ribosomal RNA was used as an internal control to quantitate the target gene expression (ΔCT).

### Cell culture and treatment

Several hepatoma cell lines (HepG2, Smmc7721, HeP3B, QGY7701, QGY7703, MHCC97L, MHCC97H, SK-HEP-1), one immortalized liver cell line (HL7702) were used in this study. HepG2, Smmc7721, HeP3B, QGY7701, QY7703, MHCC97L, MHCC97H and SK-HEP-1 cells were purchased from the Cell Bank of Shanghai (China). The HepG2 and Smmc7721 cells were both cultured in RPMI 1640 basic medium supplemented with 10% fetal bovine serum (FBS, cat:10100–147, Gibco, Australia). The Hep3B, QGY7701, QY7703, MHCC97L, MHCC97H, and SK-HEP-1 cells were cultured in DMEM supplemented with 10% FBS. The HL7702 cells were kindly provided by Dr. Yi Cao (Laboratory of Molecular and Experimental Pathology, Kunming Institute of Zoology, Chinese Academy of Sciences) and cultured in RPMI 1640 medium supplemented with 20% FBS.

### Western blot analysis

Protein samples were prepared with Fastprep-24 Sample Preparation equipment (MP, USA). Primary antibodies were obtained from Cell Signaling Technology and diluted 1:1,000 in 5% bovine serum albumin (BSA). Secondary antibodies were purchased from Earthox (Cat: E030120–01) and used at a 1:3,000 dilution. The membranes containing protein blots were incubated in blocking buffer (5% non-fat milk) for 1 hr at room temperature before the primary antibody addition. TBS (tris buffered saline) supplemented with 0.5% Triton X-100 (TBST) was used as the washing buffer.

### Immunohistochemistry (IHC)

Tissue specimens were dissected from the patients, rinsed with Phosphate Buffered Saline (PBS) to clear the blood and fixed in 10% neutralized buffered formalin (NBF) overnight. The tissues were embedded in paraffin and cut into 4-μm-thick sections. Primary antibodies specific to Notch3 and β-catenin were obtained from Cell Signaling Technology, and the secondary antibody was purchased from Earthox (Cat: E030120–01). The sections were processed for hematoxylin and eosin staining and IHC. The detailed protocols for the IHC procedures were obtained from Cell Signaling Technology and were strictly followed.

### RNAi interference

An siRNA-mediated depletion of Notch3 was applied to alter the Notch3 expression profile of the hepatoma cell line QGY7701 because of the increased expression of Notch3 in these cells. An siRNA-Notch3 gene knockdown kit was purchased from JIMA Biotechnology Company (Shanghai, China). There were four pairs of oligos designed to target Notch3 in this kit. First, we screened the efficacy of the Notch3 knockdown and selected two excellent pairs (1# and 4#) for the following experiments. We used Lipfectamine RNAiMAX (Lifetechnology, USA) to carry the siRNA in and 20pmol siRNA were transfected to each 24-well plate. This experiment was carried out according the manufactory's protocol. For stable knockdown of Notch3, 4# siRNA and control si-RNA were respectively constructed into a lenti-shRNA vector system and stable-depleted Notch3 cells were obtained after 7days puromycin selecting.

We used Lipofectamine 3000 reagent to transfect 20 pmol of siRNA for each 24-well plate. This experiment was carried out according the manufacturer's protocol (Life Technology, USA).

### Flow cytometry analysis

Cells were collected and washed twice with PBS and fixed in 4% formaldehyde for 10 min at room temperature. The fixing solution was removed by centrifugation, and the cells were resuspended in 90% methanol for permeabilization. The cells were washed twice in washing buffer (0.5% BSA in PBS). The cells were then resuspended in 100 μl of primary antibody at a dilution of 1:100 in washing buffer and incubated for 1 hr. After washing, the cells were resuspended in an Alexa Fluor-488-conjugated secondary antibody at a dilution of 1:200 in washing buffer for 30 min at room temperature. After centrifugation, the cells were washed once, resuspended in 0.5 ml of PBS and analyzed with a flow cytometer.

### Fluorescence-activated cell sorting (FACS)

The antibodies used for the FACS analysis (APC-conjugated Notch3 antibody: FAB6870A; Isotype antibody: IC002A) were obtained from R&D Systems, Inc. Cells were collected by adding 0.25% trypsin supplemented with 0.5 mM EDTA. Aliquots of cells (1 × 10^6^ cells/100 μl) were placed into FACS tubes, and 10 μl of the APC-conjugated antibody was added to each tube; the tubes were then incubated for 30 minutes at room temperature in the dark. The negative control was incubated with the APC-conjugated isotype antibody. Notch3-high A total of 3 tubes of cells were sorted into two groups, Notch3-high and Notch3-low, which were cultured for the following experiment. The two groups of Notch3-positive cells were analyzed via flow cytometry at days 7 after sorting. The details of the FACS experiment can be found in the protocol provided by R&D Systems, Inc on the company's web site.

### Aldehyde dehydrogenase (ALDH) assay

Some studies have demonstrated that ALDH is a cancer cell stemness marker of hepatoma cells and that a high expression of ALDH is an indicator of a poor prognosis for HCC patients. We measured the ALDH activity in the high-Notch3-expressing cell population and the Notch3-knockdown cells. The ALDH detection kit was obtained from Stemcell Technologies (cat# 01700, Canada). Cells were prepared at a concentration of 1×10^6^ cells/ml and aliquoted into the DEAB control (DEAB is an inhibitor of ALDH) and test tube for each sample. We strictly adhered to the instructions provided by the manufacturer.

### Dual luminescence assay

The entire 1,260-bp length of the promoter of β-catenin (1,034 bp upstream of the transcription start site and 225 bp downstream of the transcription start site) was cloned from the human genome and inserted into the PG04 vector (Genecopoeia, Inc.) upstream of a secreted Gaussia luciferase (Gluc) gene. The secreted alkaline phosphatase (SEAP) gene was used as the internal control, which was integrated into the same vector. The vector was transfected into the cells, the medium was collected, and the activities of Gluc and SEAP were measured using the Secrete-Pair^TM^ Dual Luminescence Assay Kit. We strictly complied with the User Manual provided by the manufacturer.

### Colony formation assay

Cells in the logarithmic phase of growth were digested with 0.25% trypsin and percussed into a single cell. A total of 1000 cells were seeded into each well of a 6-well plate and cultured for seven days. The medium was then removed, and the cells were washed twice with PBS. After removal of the PBS, the cells were stained with crystal violet. The result was photographed with a camera, and the number of clones was determined using ImageJ software.

### Case information collection

The pathological information for all the cases was collected from hospital documents that included detailed diagnostic results [alpha-fetoprotein (AFP) levels, differentiation grade] and basic patient information (sex, age, etc.). The differentiation grade was estimated by an experienced pathologist. Differences in the cell morphology, mitotic state and ki67 index between the tumor and normal liver tissues were used to evaluate the cell differentiation grade.

### Statistical analysis

Statistical analyses were performed using GraphPad version 5.0 software. Student's *t*-tests were performed to compare differences between two groups. Pearson correlation analyses were used to examine the correlation of two parameters. A *p* value < 0.05 was considered statistically significant.

## SUPPLEMENTARY FIGURE


