# Supplementary material for: Notch3 functions as a regulator of cell self-renewal by interacting with the β-catenin pathway in hepatocellular carcinoma
Source: Oncotarget. 2015 Feb 11;6(6):3669–79. doi: 10.18632/oncotarget.2898 (PMC4414145; doi:10.18632/oncotarget.2898)
Supplement: Supplementary file 1 [file oncotarget-06-3669-s001.pdf]

SUPPLEMENTARY FIGURE

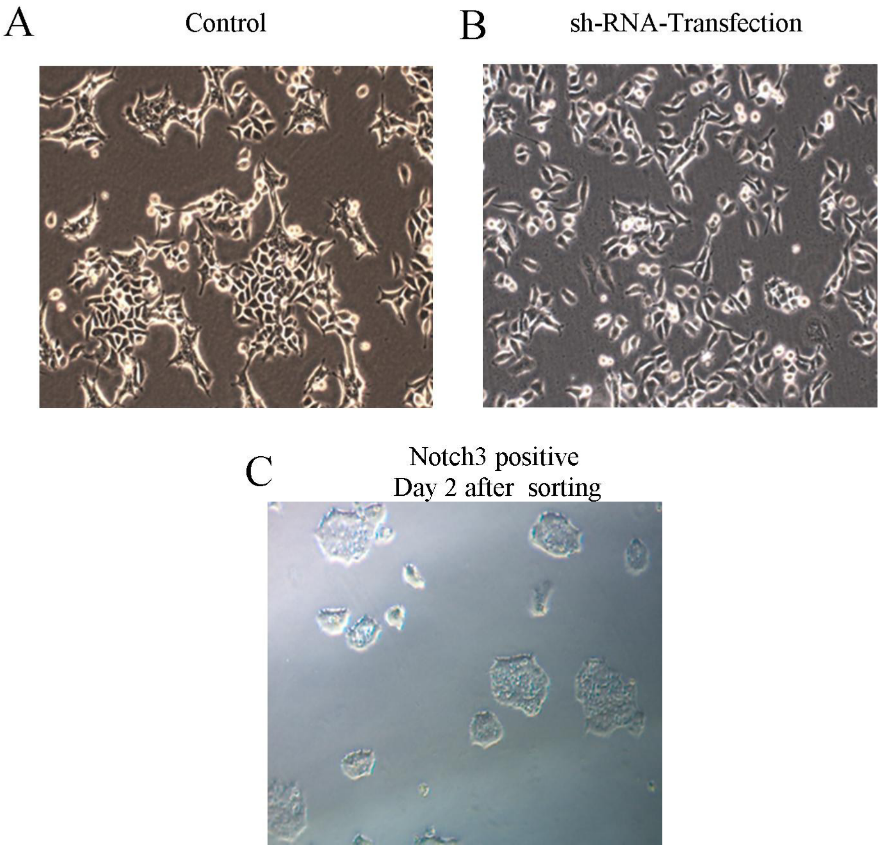

Supplementary Figure S1: The QGY7701 cells before the lenti-shRNA transfection **(A)**; QGY7701 cells after transfection with the lenti-shRNA **(B)**; QGY7701 cells were sorted with FACS and cultured for 2 days **(C)**.
